# Friction Induces Anisotropic Propulsion in Sliding Magnetic Microtriangles

**DOI:** 10.1021/acs.nanolett.2c02295

**Published:** 2022-09-05

**Authors:** Gaspard Junot, Sergi G. Leyva, Christoph Pauer, Carles Calero, Ignacio Pagonabarraga, Tim Liedl, Joe Tavacoli, Pietro Tierno

**Affiliations:** † Departament de Física de la Matèria Condensada, Universitat de Barcelona, 08028 Barcelona, Spain; ‡ Universitat de Barcelona Institute of Complex Systems (UBICS), Universitat de Barcelona, 08028, Barcelona, Spain; § Faculty of Physics and Center for Nano Science, Ludwig-Maximilians-Universität, Geschwister-Scholl-Platz 1, München 80539, Germany; ∥ Institut de Nanociéncia i Nanotecnologia, Universitat de Barcelona, 08028, Barcelona, Spain; ⊥ CECAM, Centre Européen de Calcul Atomique et Moléculaire, École Polytechnique Fédérale de Lausanne (EPFL), Batochime, Avenue Forel 2, 1015 Lausanne, Switzerland; # Departament de Física de la Matèria Condensada, Universitat de Barcelona, 08028, Barcelona, Spain

**Keywords:** Active Colloids, Micromotors, Magnetism, Soft-lithography, Shape-anisotropy

## Abstract

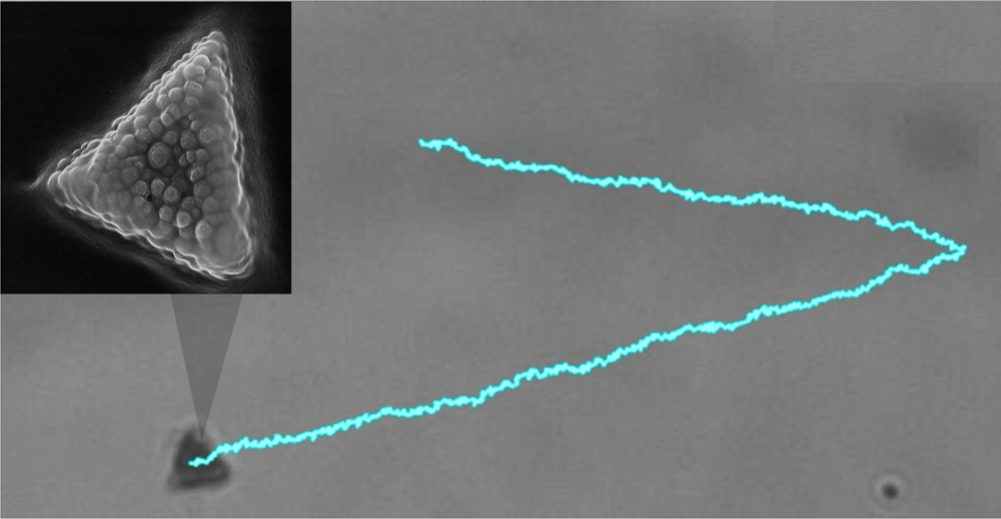

In viscous fluids, motile microentities such as bacteria or artificial swimmers often display different transport modes than macroscopic ones. A current challenge in the field aims at using friction asymmetry to steer the motion of microscopic particles. Here we show that lithographically shaped magnetic microtriangles undergo a series of complex transport modes when driven by a precessing magnetic field, including a surfing-like drift close to the bottom plane. In this regime, we exploit the triangle asymmetric shape to obtain a transversal drift which is later used to transport the microtriangle in any direction along the plane. We explain this friction-induced anisotropic sliding with a minimal numerical model capable to reproduce the experimental results. Due to the flexibility offered by soft-lithographic sculpturing, our method to guide anisotropic-shaped magnetic microcomposites can be potentially extended to many other field responsive structures operating in fluid media.

Shape anisotropy plays an important role in magnetic systems, since it creates a demagnetizing field and a preferred direction for magnetization.^1^ Anisotropy is also an intrinsic property of many biological systems, from elongated bacteria^2^ to epithelial cells in tissue sheets^3^ and vertebrate bodies,^4^ while being of crucial importance for the behavior of nanoscale systems.^5−9^ In colloidal science, shape anisotropy affects the fundamental behavior of microscopic particles dispersed in liquid media, from Brownian motion^10^ to crystal frustration,^11^ packing^12,13^ and glassy behavior.^14,15^ Anisotropic colloids can be easily manipulated via external fields,^16^ and their controlled motion has been used in several applications to date, such as probing the viscoelastic properties of complex fluids,^17−19^ or stirring and mixing liquids in confined microfluidic systems.^20−22^ For self-propelling particles systems,^23^ where injected or environmental energy is directly converted into directed motion, the anisotropic shape may induce curved trajectories,^24,25^ or be responsible for emergent collective behaviors different from those of isotropic ones.^26−28^

Here we realize isosceles magnetic microtriangles and demonstrate their propulsion in a viscous fluid when subjected to a time-dependent, conical precessing field. Depending on the field parameters, i.e. the amplitudes and driving frequency, we observe three distinct regimes of motion, where the triangles perform rolling or tumbling-like dynamics, and a sliding mode characterized by an average static planar orientation. In the latter case, the triangles hold their surface quasi parallel to the bounding wall and we show that, when the direction of the magnetic moment does not coincide with the long side of the triangle, friction asymmetry between the two short sides induces a nonzero transversal drift. Under such conditions, one can transport the triangle along different directions across the plane, even performing closed orbits. In contrast, such trajectory reduces to a line when the magnetic moment is aligned with the long side. We explain these observations with a minimal simulation scheme which considers three linked ferromagnetic particles close to a stationary bounding wall, avoiding the complexity of considering a continuous triangular shape. We demonstrate with our simulation model that such transverse drifts take place due to the coupling of the shape anisotropy and magnetic misalignment of the triangle moment with respect to the symmetric axes. Thus, our results show how magnetic misalignment can lead to new microswimmers capabilities including the realization of very specific trajectories and their operations near solid surfaces.

The ferromagnetic microtriangles are realized by filling polydimethylsiloxane molds with a suspension of silica magnetic nanoparticles (400 nm diameter) dispersed in a monomer matrix, see Figure 1a,b and Section S1 in the Supporting Information (SI) for more details. The triangles are ∼1 μm thick and isosceles, with two equal sides of length 5.1 μm, and a longer one equal to 6.1 μm. After cross-linking the monomer and extracting the triangles from the mold, Figure 1c, we disperse the obtained particles in highly deionized water, and insert the solution in a glass microchannel of height 100 μm and width ∼2 mm. The triangles sediment close to the bottom of the channel due to density mismatch, and there they display small thermal fluctuations in both the translational and orientational degrees of freedoms.

**Figure 1:**
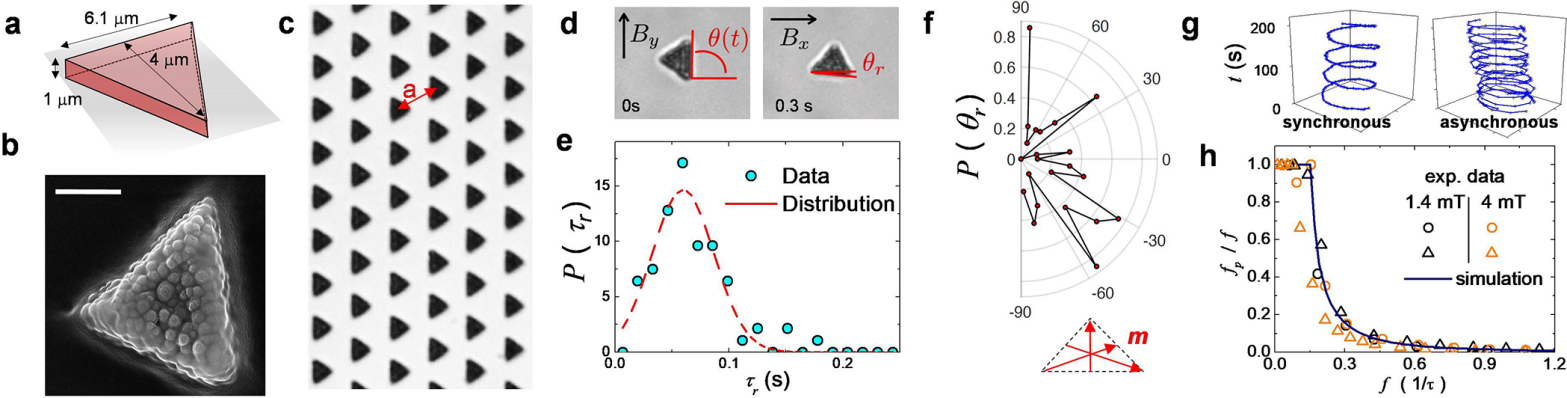
(a) Schematic of a ferromagnetic microtriangle with the corresponding sizes. (b) Scanning electron microscope image showing the embedded ferromagnetic nanoparticles (size 400 nm), scale bar is 2 μm. (c) Optical microscope image of an array of microtriangles before its extraction, lattice constant is *a* = 12.4 μm. (d) Microscope images showing the reorientation when a static field along the *ŷ*-direction (*B*_*y*_ = 1 mT) is suddenly switched along the *x̂* direction (*B*_*x*_ = 1 mT). See also SI Video S1. (e) Distribution *P* (τ_r_) of the relaxation time τ_r_ of the microtriangles measured respect to the *x*-axis. Symbols are experimental data, continuous line is a Gausan function with mean ⟨ τ_*r*_ ⟩ = 60 ms. (f) Top: Angular distribution *P* (θ_*r*_) of the reorientation angle θ_*r*_ . Bottom: schematic showing the three main directions of ***m*** within a microtriangle. (g) Position in the  plane versus time (vertical axis) of the tip of one magnetic triangle under a rotating magnetic field (amplitude *B*_0_ = 1.4 mT) in the synchronous (left, driving frequency *f* = 1 Hz) and asynchronous (right, *f* = 7 Hz) regimes. (h) Normalized rotational frequency of the triangle *f*_p_ /*f* versus *f* for two different triangles (circles and triangles) and field amplitudes (black and orange). The frequency is measured with respect to the reduced time τ (see text), the continuous line results from numerical simulations.

Free triangles display magnetic attraction due to the presence of a permanent magnetic moment ***m***. To measure the amplitude and direction of ***m*** within these structures, we investigate the triangle reorientation under a static field *B*_*y*_ = 1 mT. First, the field is applied along one direction (*ŷ*-axis) and then is suddenly switched along the perpendicular one (*x̂*-axis), see Figure 1d and SI Video S1. One can describe this reorientation in terms of a balance between the applied magnetic torque τ_m_ = |***m*** × ***B***| = *mB* sin θ with the viscous one . Here θ describes the angle between the direction of ***m*** within the triangle and the applied field, and ζ_r_ is the rotational friction coefficient. In the overdamped limit, **τ**_m_ + **τ**_v_ = 0 and the resulting solution, tan(θ/2) = exp(−*t*/τ_r_) determines the relaxation time, τ_r_ = ζ_r_ /(*mB*). As shown in Figure 1e, after studying the reorientation of 73 triangles, we find that the distribution of relaxation times *P* (τ_r_) is nearly Gaussian, centered around a mean value of ⟨τ_r_⟩ = 60 ms with a standard deviation  ms. Using ζ_r_ ∼ 8*πηV*_t_, with η = 10^–3^ Pa·s the viscosity of water and *V*_t_ = 1.22 × 10^–17^μm^3^ the triangle volume, we obtain a permanent moment of *m* = 6.4 × 10^–21^ A m^2^.

Further, the reorientation experiments provide information on the direction θ_*r*_ ∈ [−π/2, π/2] subtended by the magnetic moment with the long side of the triangle, which in turn allows to identify the corresponding direction of ***m*** within the triangle. As shown in Figure 1f, the permanent moment is oriented along three main directions, θ_***r***_ = −45°, 45° and 90°, see also the schematic at the bottom of Figure 1f. As we show below, depending on the location of ***m*** one can obtain different types of trajectories by changing the field parameters.

The magnetic properties of the microtriangles can alternatively be characterized by monitoring its response to a circularly polarized, in plane rotating magnetic field,  being *f* the driving frequency and *B*_0_ = *B*_*x*_ = *B*_*y*_ the field amplitude. The rotating field applies a magnetic torque **τ**_m_ which induces a rotational motion around a central axis. One can identify two dynamic regimes that emerge when tracking the position of one tip of the triangle as a function of time, Figure 1g. Below a critical frequency *f*_*c*_ the triangle rotates synchronously with the driving field, the phase-lag angle φ between ***m*** and ***B*** is constant and the rotational frequency *f*_p_ = *f*. In contrast, for higher frequencies, *f* > *f*_c_ the motion becomes asynchronous and the spatiotemporal plot displays small kinks where *m* loses its phase with *B* and *f*_p_ decreases as *f* increases. Such regime can be described in terms of the Adler equation,^29^ which gives in the deterministic limit . Here *f*_c_ = *f*_c_(ζ_r_, ***m***, ***B***) and thus triangles with different magnetic moments ***m*** will be characterized by a different critical frequencies. However, all data can be rescaled by plotting *f*_p_/*f* versus the driving frequency measured in terms of a reduced time, τ = 1/(2*πf*_c_ ). This reduced time compares the magnetic torque with the viscous one. When *f* (1/τ) ≳ (2π)^−1^, the viscous torque resistance is larger than the magnetic one, which gives rise to the asynchronous regime. Figure 1h shows *f*_p_ /*f* against *f* /τ for two different types of triangles (circles and triangles) and at two amplitudes of the rotating field, *B*_0_ = 1.4 and 4 mT. This scaling also leads to excellent quantitative agreement with numerical simulations of a minimal model of the microtriangles, more details will be given later.

We induce propulsion of the microtriangle in water by applying a magnetic modulation that precesses with frequency *f* around an axis parallel to the glass substrate . A field that precesses around the *ŷ* -axis is given by
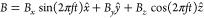
1This type of magnetic modulation has been used in the past as a convenient means to transport other types of anisotropic objects, including paramagnetic doublets,^30^ ribbons,^31^ or composite particles.^32,33^ When this modulation is applied to a microtriangle, it tries to align its moment with the precessing field, which would induce a conical rotation, similar to a gyroscope spinning. However, due to the complex shape of the triangle, the relative large aspect ratio (area to thickness) and the steric interaction with the bounding wall, we find three types of transport modes, depending on the different field parameters, Figure 2a. For low amplitude of the static, in-plane component *B*_*y*_ , (*B* ≲ 0.5 mT), Figure 2a left, middle, and right, the microtriangle rotates perpendicularly to the bounding wall, and it moves as a microscopic wheel, see first row of Figure 2b, Figure 2c and the SI Video S2. This transport mode is observed for a wide range of frequencies (*f* ∈ [10, 60] Hz). The triangle transport is induced by the rotational-translational coupling, resulting from the dependence of the friction with the fluid on the distance to the bounding wall.^34^ Due to the relative small thickness of the triangles, the wheel motion is usually characterized by a small translational speed of the order of ⟨*v*⟩ ∈ [0.5, 2]μm/s.

**Figure 2:**
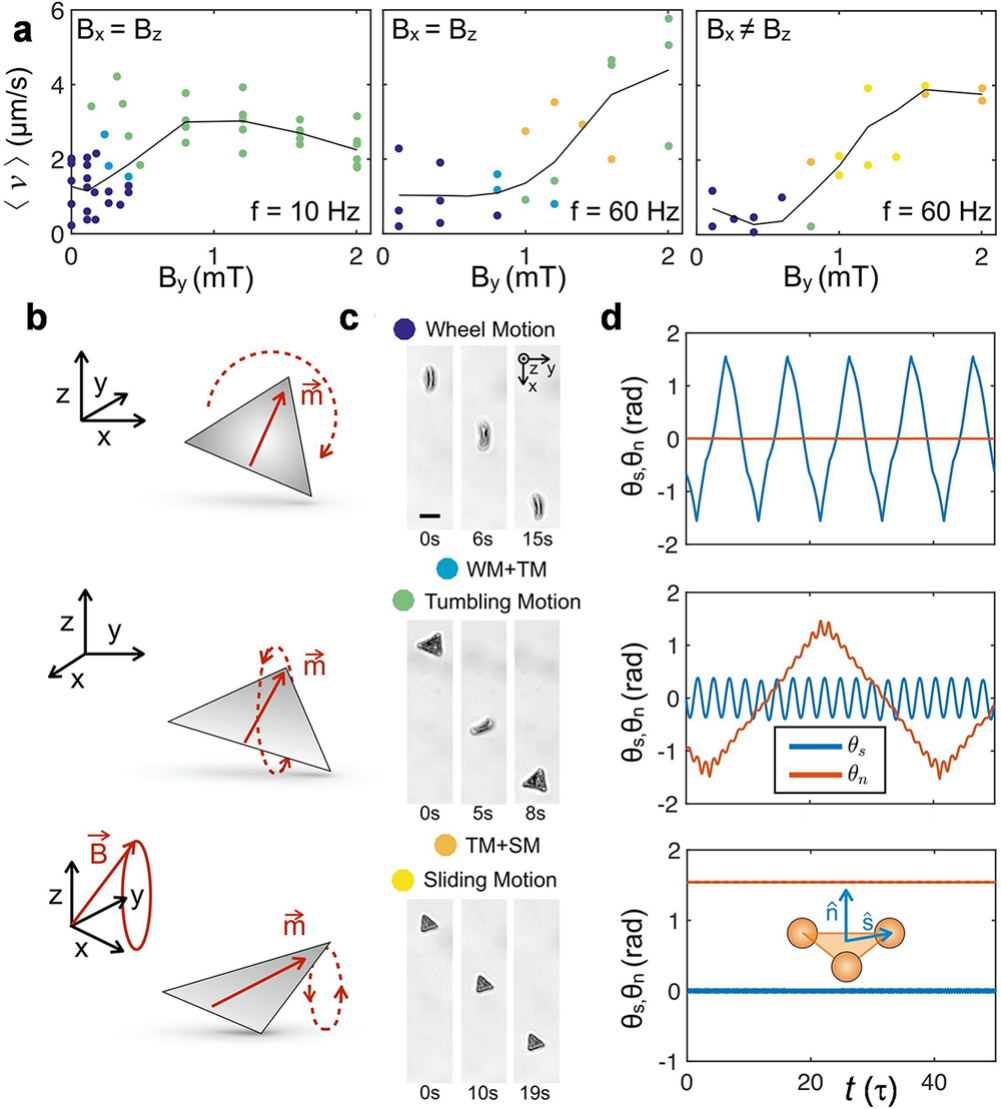
(a) Mean speed ⟨*v*⟩ with  versus static component *B*_*y*_ of the precessing field for two different frequencies *f* = 10 Hz and *f* = 60 Hz at *B*_*x*_ = *B*_*z*_ = 1.6 mT (first and second panel) and at amplitudes *B*_*x*_ = 1.4 mT and *B*_*z*_ = 1.27 mT (third panel). (b,c) Schematic (b) and sequence of images (c) taken at three different instants of times of a propelling microtriangle in the three regimes: wheel (top, *B*_*x*_ = *B*_*z*_ = 1.6 mT, *B*_*y*_ = 0 mT, *f* = 10 Hz), tumbling (middle, *B*_*x*_ = *B*_*z*_ = 1.6 mT, *B*_*y*_ = 0.32 mT, *f* = 10 Hz) and sliding (bottom, *B*_*x*_ = 1.4 mT, *B*_*z*_ = 1.27 mT, *B*_*y*_ = 1.2 mT and *f* = 60 Hz). The scale bar in the top image is 5 μm, the number of observed events are 33 for the wheel, 37 for the tumbling and 30 for the sliding mode. The corresponding videos illustrating these experimental situations are deposited as Supporting Information (Videos S2, S3, and S4). (d) Results from numerical simulations: normal (θ_n_) and vector (θ_s_) angles versus rescaled time for three situations corresponding to the experimentally observed regimes of motion. The small schematic in the bottom panel shows the modeled three particle system with the unit vectors ***n*** and ***s***.

Increasing the static component *B*_*y*_, forces the triangle to lay parallel to the bounding wall. However, for larger values of *B*_*y*_ (*B*_*y*_ ≳ 0.5 mT) the triangle still tries to follow as a whole the field modulation, and the resulting mode is a tumbling-like translation where the triangle continuously flips, second row of Figure 2b and Figure 2c. In this situation, increasing *B*_*y*_ destabilizes the in-plane rotation, and the permanent moment follows the field modulation but it features some wobbling of the microtriangle, see the SI Video S3. As shown in Figure 2a left and middle, this transport mode usually displays an higher average translational speed, ⟨*v*⟩ ∈ [2, 6]μm/s.

At high frequencies (*f* = 60 Hz) and large values of *B*_*y*_ (*B*_*y*_ ≳ 0.5 mT) and for an elliptically polarized field (*B*_*x*_ ≠ *B*_*z*_), we find that the tumbling mode transits to a surfing like propulsion, where the microtriangle is observed to translate without flipping, with an intermediate speed of ⟨*v*⟩ ∈ [2, 4] μm s^–1^, third row of Figure 2b and Figure 2c. By carefully analyzing the experimental videos, we observed that in this mode the microtriangle shape laid almost parallel to the bounding plane while displaying a fast rotational movement of the tips. These rotations have a very small amplitude, that impede to characterize them experimentally and resolve the full three-dimensional dynamics of the tips. Instead, we have used numerical simulations (details are given later) to clarify the mechanism of motion in this regime. We found that the rotations of the tips produce unequal displacements along and perpendicular to the bounding wall, which induce asymmetric dissipations capable to break the time reciprocity of the fluid flow at low Reynolds number.^35^ As shown in the SI Video S4, the microtriangles literally surf on top of the plane displaying a small wobbling. The orientation θ_*r*_ of the magnetic moment ***m*** with respect to the long triangle side varies from triangle to triangle and so does the orientation of the long triangle side with respect to the transverse direction (*ŷ*-axis). In particular, when θ_r_ = 0°, *x̂* is an axis of symmetry of the triangle whereas when θ_r_ ≠ 0° it is not. Thanks to these three modes, a triangle can adapt its locomotion to the environment. In an open environment, one can use the fastest mode (tumbling). However, when required to pass through a small orifice or pore, one can easily switch to the wheel or sliding modes which could enable the triangle to pass through these constrictions.

To confirm the experimental observations, we have developed a numerical model to gain insight in the mechanisms of the different transport modes. We represent the microtriangle as three beads, *i* = 1, ..., 3 of equal mass *m* and located at a fixed distance away from each other. The equation of motion for each particle follows
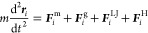
2The first term on the right side, ***F***_*i*_^m^, accounts for the net force acting on bead *i* as a result of the constraint that keeps the three beads at constant separation from each other, and to the torque due to the coupling between the magnetic moment of the microtriangle ***m*** (aligned with a prescribed axis rigidly fixed to the triangular plane) and the external magnetic field ***B***. ***F***_*i*_^g^ corresponds to the gravitational force, while ***F***_*i*_^LJ^ accounts for the steric interactions between the beads and the solid bounding wall. Finally, ***F***_*i*_^H^ denotes the force acting on the bead *i* due to hydrodynamic interactions. These forces are described in detail in the Section S2 in SI. This minimal model captures the essential mechanisms leading to rectification and thus net transport, which emerges from the coupling between the object geometry, the symmetry of the external driving and the plane mediated hydrodynamic interactions.

As shown in the small scheme at the bottom of Figure 2d, to characterize the three regimes of motion we define two unit vectors, ***n*^** and ***s*^** which define the direction perpendicular to plane of the triangle and from the center to one of the three particles, respectively. Thus, we describe the three translating modes in terms of the angles  and . For the wheel motion (top panel in Figure 2d) θ_*n*_ remains constant and the three-particle system performs rolling only in the  plane, with θ_*s*_ periodically varying within the range [−π, π] similar to the propulsion of magnetic rollers.^36^ The tumbling transport (middle panel in Figure 2d) features periodic oscillations of both angles θ_n_ and θ_s_: The θ_s_ conical precession produces a slow rotation of θ_n_, which periodically flips the microtriangle. The propulsion by flipping of the microtriangle is analogous to the motion of actuated rotors under the effect of a conical precessing field.^37^ Finally, the last panel of Figure 2d corresponds to the surfing like transport. The simulations show that the ratio between the gravitational attraction and the magnetic force plays a key role in avoiding the flipping of θ_n_, stabilizing the average planar oscillations when the triangle slides. This motion is characterized by almost constant values of both angles with small oscillations. The simulations allow to visualize the bead trajectories which represents the triangle tips. Small and fast asymmetric oscillations are observed for each tip in each period, resulting in a net propulsion, see SI Video 9.

The model also allows a deep exploration of the parameter space which unveils the different degrees of freedom that allow propulsion in the sliding mode. In particular, for a microtriangle with θ_*r*_ = 90°, Figure 3 displays how θ_s_ varies parametrically as a function of , which corresponds to the trajectory where the vertex *ŝ* points to. As the static field component *B*_*y*_ increases, both the rectification velocity of the sliding triangle and the area contained by the corresponding trajectory decrease, and eventually the trajectory does not contain a finite area, corresponding to the regime where the triangle does not slide. Now the tips’ oscillations are parallel to the boundary wall surface with a vanishing area. Hence, the parallel and perpendicular motion of the triangle vertex in the presence of the solid bounding wall provide the two independent degrees of freedom required by Purcell scallop theorem to break the time reversal symmetry and produce a translational motion.^35^

**Figure 3:**
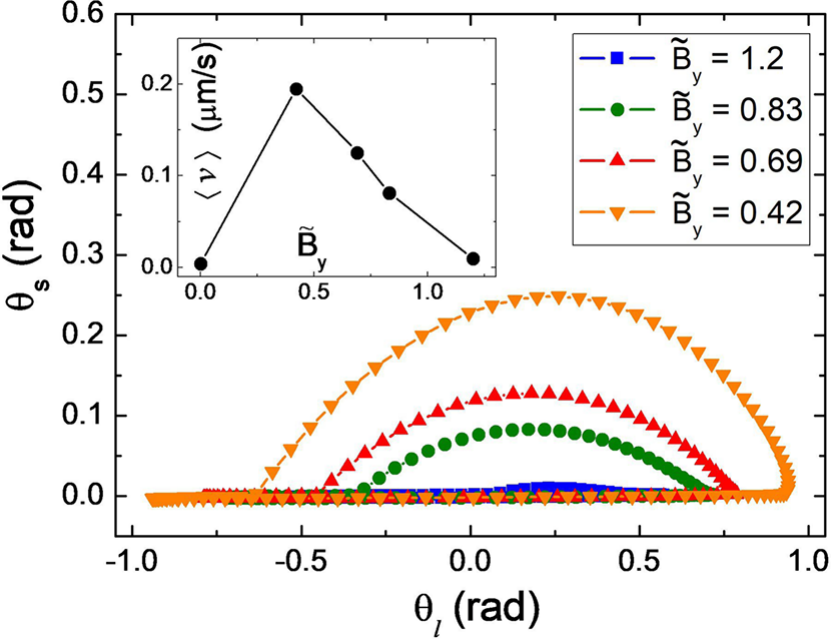
Trajectories of the angle θ_s_ as a function of θ_l_, for a simulated microtriangle with θ_r_ = 90°. Here  refers to the constant component of the magnetic field normalized by the radius of the rotating field, . The inset shows the average translational velocity of each trajectory. The frequency is set to *f* = 2.56 Hz. In this specific set of simulations, *ŝ* is parallel to the magnetization.

We now focus on the sliding mode, where the microtriangle translates almost parallel to the close bounding wall. In this regime we find that microtriangles characterized by a permanent moment θ_*r*_ ≠ 0, exhibit a net propulsion along the axis of precession (*y* axis) in addition to the motion along the perpendicular direction. As shown in Figure 4a, see also VideoS5 in the Supporting Information, this effect is robust, and reproducible, and can be used to rectify the motion of sliding triangles to bring them to any point of the plane by simply switching the chirality of the rotating field (here inverting *B*_*x*_) and the static field *B*_*y*_. In contrast, microtriangles whose magnetization is parallel to their long side (θ_r_ = 0°) do not display such asymmetric friction and the corresponding transversal drag, Figure 4b. Consequently, those triangles can only be driven along a line (here the *x̂*-axis). As shown in Figure 4c, we observe the same behavior in simulation i.e triangles exhibit transversal motion only when θ_*r*_ ≠ 0. Magnetic misalignment allows for each set of magnetic field configurations to produce a different orientation of the tips’ oscillations with respect to the laboratory frame, leading to the four transversal directions, as can be observed in SI Video S6. The sliding propulsion mode that we report does not involve complete rotations of the micro-object. In this case, the rectification of its motion into net displacement requires both the anisotropy of friction due to the presence of the solid wall and, at least, two degrees of freedom to define the particle configuration. Hence, we expect that any anisotropic object will, generically, be able to slide under the appropriate external actuating field. For example, a disk can exhibit wheel, tumbling and sliding. However, the sliding propulsion will have the same direction as the wheel and tumbling motion, depending only on the chirality of the magnetic field. This is because there cannot be misalignment in a planar magnetic moment contained in a disk. Hence, the degree of anisotropy has a strong impact in the possibility to manipulate and control the direction of motion of the object.

**Figure 4:**
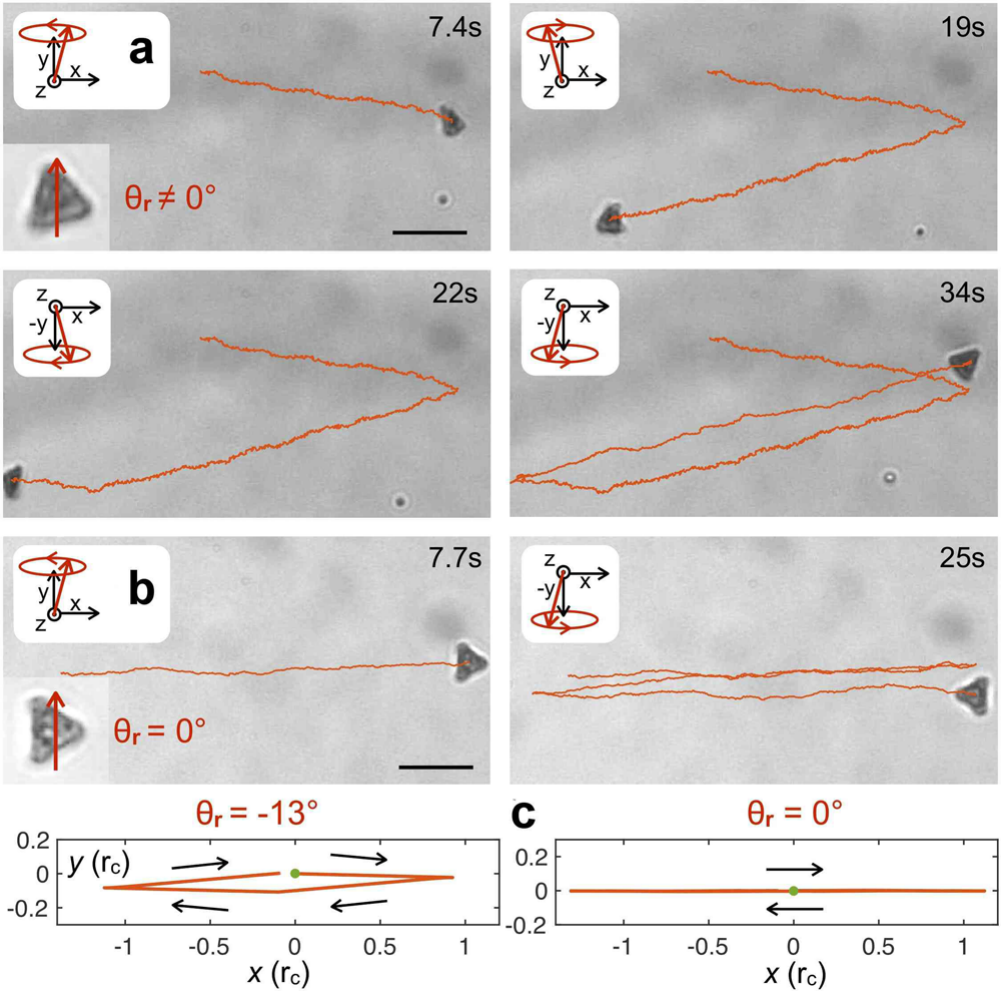
(a) Sequence of images showing a two-dimensional trajectory of a microtriangle in the sliding regime when the permanent moment is inclined with respect to its long side (θ_r_ = −17°). The change in the horizontal transport direction (*v*_*x*_ → – *v*_*x*_) is realized by inverting the chirality of the rotating field (*B*_*x*_ → – *B*_*x*_), while the change in the vertical direction is obtained by inverting the static component (*B*_*y*_ → – *B*_*y*_). The corresponding video is SI Video S3. (b) One dimensional trajectory showing the back and forward motion of a sliding microtriangle with symmetric orientation of the two short sides along the transport direction (θ_r_ = 0°). (c) Corresponding results from numerical simulations of a sliding microtriangle for θ_r_ = −13° (left) and θ_r_ = 0° (right).

At large area fractions, our ferromagnetic microtriangles can interact and assemble due to dipolar forces. As shown in the top inset of Figure 5a, already in the absence of any applied field, the particles tend to aggregate forming linear chains where the internal orientation of the individual triangles depends on the orientation of their permanent moments. Once they adopt an elongated structure, the triangles display weak thermal fluctuations and the structure is practically fixed, but they can be readily transported and redispersed in the water via an external field. For example, Figure 5a and the corresponding SI Video S7, shows the propulsion of the chain when it is subjected to a precessing field. The particles show a relative displacement advancing one with respect to the other during a field cycle, which lead to fluctuations along the *y*-position. In contrast, they tend to keep their separation distance constant, as shown by the bottom inset (*x*-position). Thus, one can translate the magnetic chain at a constant speed, and their collective motion could be used to transport other non magnetic cargoes dispersed in the fluid medium.

**Figure 5:**
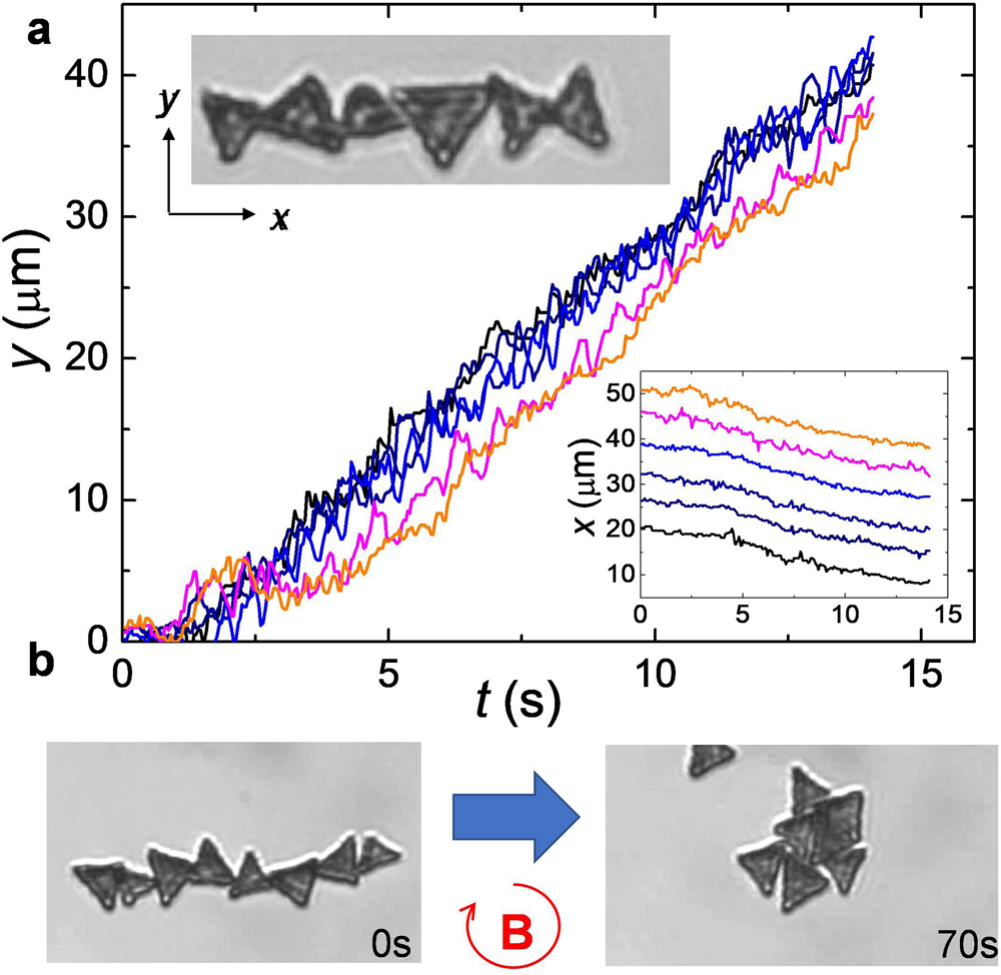
(a) Position versus time of the center of mass of 6 triangles that collectively translate via the tumbling mode at a constant speed ⟨*v*_*y*_⟩ = 3.1 μms^–1^. The applied precessing field has amplitudes *B*_*z*_ = *B*_*x*_ = 1.6 mT and *B*_*y*_ = 1.22 mT (static field) and frequency *f* = 10 Hz. Top inset displays a microscope image of the initial assembly (*B* = 0), see also SI Video S7. Bottom inset shows the transversal trajectory with a constant separation distance between the particles. (b) Microscope image showing the initial (0s) and final (70s) configuration of 8 triangles that are assembled in a compact structure due to an in-plane rotating magnetic field with *f* = 10 Hz and *B*_*x*_ = *B*_*y*_ = 1 mT.

Apart from collective transport, the magnetic triangles could be assembled in more compact structures, starting from a linear aggregate. This feature is demonstrated in Figure 5b, where the microtriangles are subjected to an in-plane, circularly polarized rotating field. The rotating field creates a torque on the particles and induce time-averaged attractive dipolar interactions.^38^ Such compact structure forms due to the competition between dipolar forces and excluded volume, while assemble the particles to reduce the free space thus maximizing packing. We note that the assembly of few microtriangles is the starting point to investigate the field-induced aggregation of more complex structures that can be easily designed with our lithographic technique.

In conclusion, we have demonstrated that lithographically made soft magnetic microtriangles display a rich series of transport modes when subjected to a conically precessing magnetic field. We find that, depending on the field parameters, these complex particles may either translate as microwheel, tumble or even display a surfing like dynamics where they slide close to the bounding wall. In the sliding mode, we find that anisotropy in friction and magnetic misalignment may be used to generate a transversal particle motion, and the microtriangle can be driven across the full plane by switching the static component of the applied field and the field chirality. Those different modes enable the triangle to adapt its locomotion to different situations, giving the triangle an advantage with respect to more simple isotropic particles. All these dynamical modes can be explained by considering a simple model of three linked ferromagnetic spheres interacting with a bounding plane. We finally stress that transport of isotropic magnetic colloids and their collective dynamics have been matter of much research so far. However, using particles with complex shapes may further unveil novel transport modes which could be used to create more complex functional operations in fluid based applications. We have demonstrated this concept with a microtriangle, but our results are rather general, as any anisotropic shaped object with a magnetic misalignment could result in a sliding propulsion with different transversal motions.

## Supporting Information Available

More details on experimental system and numerical simulation (PDF)
Videoclip illustrating the reorientation dynamics of two magnetic microtriangles initially aligned by a static field of amplitude 1 mT along the vertical (*y*) direction which is subsequently switched along the horizontal (*x*) direction (AVI)
Videoclip illustrating the wheel motion of a magnetic microtriangle which is driven first towards top and later towards bottom by inverting the chirality of the precessing field (AVI)
Tumbling motion of a magnetic microtriangle which is driven first toward top and later toward bottom by inverting the chirality of the precessing field (AVI)
Video showing the surfing-like propulsion of a magnetic microtriangle which is driven first toward top and later toward bottom by inverting the chirality of the precessig field (AVI)
Videoclip showing how a microtriangle performs a close trajectory by acquiring a transversal speed due to friction anisotropy (AVI)
Video showing position of the three tips of a microtriangle in the sliding mode obtained from numerical simulation (AVI)
Videoclip showing collective transport of six microtriangles initially assembled to form a chain AVI)

## Supplementary Material

nl2c02295_si_001.pdf

nl2c02295_si_002.avi

nl2c02295_si_003.avi

nl2c02295_si_004.avi

nl2c02295_si_005.avi

nl2c02295_si_006.avi

nl2c02295_si_007.avi

nl2c02295_si_008.avi
