# Supplementary material for: Friction Induces Anisotropic Propulsion in Sliding Magnetic Microtriangles
Source: Nano Lett. 2022 Sep 5;22(18):7408–14. doi: 10.1021/acs.nanolett.2c02295 (PMC9523709; doi:10.1021/acs.nanolett.2c02295)
Supplement: Supplementary file 1 — nl2c02295_si_001.pdf [file nl2c02295_si_001.pdf]

Supporting Information for
Friction induces anisotropic propulsion in sliding magnetic microtriangles

Gaspard Junot, Sergi G. Leyva, Christoph Pauer, Carles Calero, Ignacio Pagonabarraga, Tim Liedl, Joe Tavacoli, and Pietro Tierno*

E-mail: ptierno@ub.edu

Section S1: Experimental methods

Preparation of the magnetic microtriangles. PDMS molds holding microwells with triangular cross sections were fabricated using standard soft-lithographical techniques and templated by an SU8 resin as detailed in a previous work.[1]

To prepare the colloidal precursor dispersion we use superparamagnetic particles made of hematite with a silica shell (GE Healthcare, Serasil-Mag, diameter = 400nm) in a liquid monomer ethoxylated trimethylolpropane triacrylate (ETPTA, Sigma, Mn $\sim$ 428). This suspension was used as a precursor formulation to fill the microwells and make the triangles. To enhance the stability of the magnetic particles within ETPTA, the magnetic particles were first treated at room temperature at a concentration of 0.1% v/v within a $5:1$ methanol:ammonia(aq) ($10\%\mathrm{wt}$) solution with $0.5\% v/v$ 3-(tremethoxysilyl)propyl methacrylate for two days. The now treated magnetic particles were then cleaned by five cycles of centrifugation and supernatant removal with methanol before finally transferring to ETPTA at $33\% v/v$ with $4\% v/v$ of the photoinitiator 2-hydroxy-2-methyl-1-phenyl-propan-1-one (Sigma) added to the final mixture.

PDMS microwells were filled with the magnetic colloidal dispersion in ETPTA by sliding a $20\mathrm{muL}$ droplet of it over the PDMS surface by tilting the mold to $45°$. After filling, the dispersion was reticulated in the wells overnight under a 254 nm hand-held UV lamp (NU4 KL, Benda Laborgeraete).

Experimental setup. The dynamics of the propelling particles are observed using an upright light microscope (Eclipse Ni, Nikon) equipped with a Charge-Coupled Device Camera (Scout scA640-74f, Basler) and different oil immersion objective ($100\times$ and $60\times$), depending on the degree of magnification required. We mount on the microscope stage a set of custom made magnetic coils arranged to apply time dependent magnetic fields. The coils that generate rotating field are driven by a power amplifier (IMG STA-800, stage line) which is controlled via a wave generator (Aim- TTi TGA1244). We obtain a rotating magnetic field in a given plane by passing through two perpendicular coils two sinusoidal current with $90°$ phase shift. Further, a static field is obtained by using a DC power supplier (TTi El 302).

Section S2: Numerical simulation

We provide here a detailed account of the terms of Eq. **??** which determine the dynamics of the microtriangle. $\mathbf{F}_i^g = -mg\hat{\mathbf{z}}$ is the gravitational force. $\mathbf{F}_i^{LJ}(z_i)$ accounts for the steric interaction of a bead located a distance z_i above the bounding planar solid wall. The steric potential is the Weeks-Chandler-Andersen (WCA) potential, which consists in the repulsive part of the Lennard-Jones potential. To calculate $\mathbf{F}_i^m$ we assume that the magnetic torque is applied to the centre of mass of the triangle at position $\mathbf{r_{CM}}$,

$$\boldsymbol{\tau} = \sum_i^3 (\boldsymbol{r}_i - \boldsymbol{r}_{CM}) \times \boldsymbol{F}_i^m \tag{1}$$

As the torque does not induce any net force on the triangle, we add the extra constraint $\sum \mathbf{F}_i^m = 0$, which allows to rewrite Eq 1 as

$$\boldsymbol{\tau} = (\mathbf{r_1} - \boldsymbol{r}_3) \times \boldsymbol{F}_1^m + (\boldsymbol{r}_2 - \boldsymbol{r}_3) \times \boldsymbol{F}_2^m \tag{2}$$

We also impose that the torque does not produce any local tensions along the sides of the triangle. At each step, we solve numerically the system of equations by performing a lower-upper (LU) decomposition combined with a backward and a forward substitution algorithm.[2] Additionally, we ensure the separation between particles remains fixed by means of the MILC SHAKE algorithm.[3]

$\boldsymbol{F_i^H}$ accounts for the hydrodynamic interactions. In the vicinity of a stationary bounding wall, a bead moving in a viscous fluid experiences a flow generated by its own image, but also by the motion of all the other beads and their images. For Stokes flow, the fluid velocity at the position of bead i can be expressed as

$$\boldsymbol{v}_{iH} = \Delta\boldsymbol{\nu}_i \mathbf{F}_i^t + \sum_{j \neq i} \boldsymbol{G}(\boldsymbol{r}_i, \boldsymbol{r}_j) \mathbf{F}_j^t \tag{3}$$

in terms of the total force each bead is subject to, $\mathbf{F}_i^t = \mathbf{F}_i^m + \mathbf{F}_i^m + \mathbf{F}_i^{LJ}$. The first term in the right side of Eq. 3 corresponds to the self interaction contribution of the particle with its own image on the stationary bounding wall. The tensor $\boldsymbol{\Delta\nu}$ captures this interaction $\boldsymbol{\Delta\nu} = \nu(\mathbf{1} - \hat{\boldsymbol{z}}\hat{\boldsymbol{z}}) + 2\nu\hat{\boldsymbol{z}}\hat{\boldsymbol{z}}$, where $\nu = -\frac{3}{16}\frac{a}{z_i}$, a is the hydrodynamic radius of the bead, and z_i its z coordinate distance to the stationary bounding wall.[4] The second term provides the cross hydrodynamic interactions between different beads: $\boldsymbol{G}(\boldsymbol{r}_i, \boldsymbol{r}_j) \equiv \boldsymbol{G}_{ij}$ which takes into account the hydrodynamic flux contribution between a bead j, its image, and bead i. Due to this velocity field, particles experience an hydrodynamic drag that can be calculated as $\boldsymbol{F}_i^H = -\boldsymbol{\gamma}(\dot{\boldsymbol{r}}_i - \boldsymbol{v}_{iH})$, so that the final expression for the force is

$$\mathbf{F}_i^H = -\gamma_0^{\parallel} \left(\hat{\mathbf{n}}\hat{\mathbf{n}} \frac{\gamma_0^{\perp}}{\gamma_0^{\parallel}} + \hat{\mathbf{p}}\hat{\mathbf{p}} \right) \left[\dot{\mathbf{r}}_i - \Delta\nu_i \mathbf{F}_i^t - \frac{1}{8\pi\eta} \sum_j \mathbf{F}_j^t \mathbf{G}_{ij} \right] \tag{4}$$

Here we have considered an asymmetric friction $\hat{\boldsymbol{\gamma}}$, which takes into account the difference in

friction of beads when they move parallel to the plane of the triangle, or perpendicular to it. The terms $\hat{\mathbf{n}}\hat{\mathbf{n}}$ and $\hat{\mathbf{p}}\hat{\mathbf{p}}$ are tensors that determine the hydrodynamic friction normal ($\hat{\mathbf{n}}$) or perpendicular ($\hat{\mathbf{p}}$) to the triangle plane. The scalars $\gamma_0^{\perp}$, $\gamma_0^{\parallel}$ denote the bead friction perpendicular and parallel to the triangle plane. This difference in friction accounts for the planar geometry of the triangle.

The tensor $\mathbf{pp}$ defines the plane of the triangle, $\hat{\mathbf{p}}\hat{\mathbf{p}} = 1 - \hat{\mathbf{n}}\hat{\mathbf{n}}$. In the limit $\gamma_0^{\perp}$=$\gamma_0^{\parallel}$, Eq. 4 reduces to the scalar form of the friction tensor for spherical beads. We consider the far field hydrodynamic coupling between beads and consider the Blake-Green expression for $\mathbf{G_{ij}}$,[?] which takes into account the hydrodynamic interaction between beads in the presence of a stationary plane at $z = 0$.

Using the characteristic length of the triangle, r_c, and the characteristic relaxation time $\tau = {\gamma_0}^{\parallel} r_c^2/|m||B|$, one can express Eq. **??** in dimensionless form as

$$\frac{t_a}{\tau}\ddot{\tilde{r}}_i = -\hat{\boldsymbol{\gamma}}\dot{\tilde{\mathbf{r}}}_i + (\tilde{\mathbf{F}}_i^m + \tilde{\mathbf{F}}_i^g + \tilde{\mathbf{F}}_i^{LJ})(\mathbf{1} + \hat{\boldsymbol{\gamma}}\Delta\boldsymbol{\nu}_i) + \frac{3}{4}\frac{a}{r_c}\sum_j \tilde{\mathbf{F}}_j^t \tilde{\mathbf{G}}_{ij} \tag{5}$$

where t_a=$\frac{m}{\gamma}$ is the inertial time, $\tilde{\boldsymbol{r}}_i = \boldsymbol{r}_i/r_c$, and $\tilde{\boldsymbol{F}}_i^m \equiv \|\boldsymbol{F}_i^m||\boldsymbol{r}|/|\boldsymbol{m}||\boldsymbol{B}|$, and the factor a/r_c compares the thickness to the size of the triangle.

The force $\tilde{\mathbf{F}}_i^g = -\xi\hat{\mathrm{e}}_z$, with $\xi \equiv r_c mg/|m||B|$ accounts for the relative magnitude of the gravitational field compared with the applied magnetic torque. A large torque compared to the triangle weight, induced by $|\vec{B}||\vec{m}|/r_c \gg mg$ implies a negligible ξ. Experimentally this parameter can only be controlled through the applied magnetic field. $\tilde{\mathbf{F}}_i^{LJ}$ accounts for the interaction of each bead with the bounding wall, and reads $\tilde{\mathbf{F}}_i^{LJ} = (r_c u_0/|m|B|)\hat{\mathbf{e}}_{\mathbf{z}}|\hat{\mathbf{r}}_{\mathbf{z}}|^{-\mathbf{13}}$, with u_0 being the strength of the steric repulsion.

Therefore, the relevant parameters of the model are a/r_c, $\frac{\gamma_0^{\perp}}{\gamma_0^{\parallel}}$, $B_y/|\vec{B}_{rot}|$, ξ, f. we tune in simulation the first three parameters, while obtain the other from the experimental system. As characteristic values, we take the hydrodynamic radius to be close to triangle thickness,$a \simeq 0.15$,$\gamma_0^{\parallel} = 1$ and $\frac{\gamma_0^{\perp}}{\gamma_0^{\parallel}} = 2$. The rest of the parameters, $B_y/|\vec{B}_{rot}|$, ξ, $f\tau$ will be varied to characterise the different dynamic regimes in simulations.

We integrate Eq. 5 using an implicit, two step Velocity-Verlet algorithm in matrix notation to deal with the coupling introduced by the tensorial friction.

Section S3: Supporting video files

With the article there are 74 videoclips as support of Figures and Main text.

- **VideoS1(.AVI)**: This videoclip illustrates the reorientation dynamics of two magnetic microtriangles initially aligned by a static field of amplitude 1mT along the vertical (y) direction which is subsequently switched along the horizontal (x) direction.

- **VideoS2(.AVI)**: This videoclip illustrates the wheel motion of a magnetic microtriangle which is driven first towards top and later towards bottom by inverting the chirality of the precessing field. The precessing field has frequency $f = 10$Hz, and amplitudes $B_x = B_z = 1.6$mT, $B_y = 0$. The video corresponds to the sequence of images at the top of Figure 2(c) of the article.

- **VideoS3(.AVI)**: Tumbling motion of a magnetic microtriangle which is driven first towards top and later towards bottom by inverting the chirality of the precessing field. The applied field has frequency $f = 10$Hz, and amplitudes $B_x = B_z = 1.6$mT and $B_y = 0.32$mT. The video corresponds to the sequence of images in the middle of Figure 2(c) of the article.

- **VideoS4(.AVI)**: Video showing the surfing like propulsion of a magnetic microtriangle which is driven first towards top and later towards bottom by inverting the chirality of the precessig field. The precessing field has frequency $f = 10$Hz, and amplitudes $B_x = B_z = 1.6$mT and $B_y = 0.32$. The video corresponds to the sequence of images at the bottom of Figure 2(c) of the article.

- **VideoS5(.AVI)**: This videoclip shows how a microtriangle performs a close trajectory by acquiring a transversal speed due to friction anisotropy. The center of mass position is superimposed to the image. The video has been speed-up $10\times$. The precessing magnetic field

has frequency $f = 10$Hz, and amplitudes $B_x = B_z = 1.6$mT and $B_y = 0.32$. The video corresponds to the sequence of images in Figure 3(a) of the article.

- **VideoS6(.AVI)**: Video showing the position of the three tips of a microtriangle in the sliding mode obtained from numerical simulation. The used parameters are $f = 0.5/\tau$ being τ the reduced time (see text), $B_y/B_{rot} = 1.25$ where $B_{rot} = |B_x + B_z|$, and $B_x = 1.0$, $B_z = 1.0$ where all field amplitudes have been made adimensional.

- **VideoS7(.AVI)**: This videoclip shows the collective transport of 6 microtriangle initially assembled to form a chain. The precessing magnetic field has frequency $f = 10$Hz, and amplitudes $B_x = B_z = 1.6$mT and $B_y = 1.22$mT. The video corresponds to the sequence of images in Figure 5(a) of the article.

References

(1) J. W. Tavacoli, P. Bauer, M. Fermigier, D. Bartolo, J. Heuvingh, O. du Roure, The fabrication and directed self-assembly of micron-sized superparamagnetic non-spherical particles. *Soft Matter* **2013**, 9, 9103.

(2) W. H. Press, S. A. Teukolsky, W. T. Vetterling, B. P. Flannery *Numerical Recipes in C*, 2nd ed.; Cambridge University Press: Cambridge, USA, **1992**.

(3) A. G. Bailey, C. P. Lowe, A. P. Sutton, Efficient constraint dynamics using MILC SHAKE. *J. Comp. Phys* **2008**, 227, 8949–8959.

(4) E. M. Gauger, M. T. Downton, H. Stark, Fluid transport at low Reynolds number with magnetically actuated artificial cilia *Eur. Phys. J. E* **2009**, 28, 231-242.

(5) J. R. Blake, A note on the image system for a stokeslet in a no-slip boundary. *Mathematical Proceedings of the Cambridge Philosophical Society* 1971, 70, 303–310.
